# Functionally distinct smiles elicit different physiological responses in an evaluative context

**DOI:** 10.1038/s41598-018-21536-1

**Published:** 2018-03-01

**Authors:** Jared D. Martin, Heather C. Abercrombie, Eva Gilboa-Schechtman, Paula M. Niedenthal

**Affiliations:** 1 University of Wisconsin-Madison, Department of Psychology, 1202 W. Johnson St., Madison, WI 53706 United States; 2 University of Wisconsin-Madison, Department of Psychiatry, 6001 Research Park Blvd, Madison, WI 53719 United States; 3 Bar-Ilan University, Department of Psychology, Ramat-Gan, 52900 Israel

## Abstract

When people are being evaluated, their whole body responds. Verbal feedback causes robust activation in the hypothalamic-pituitary-adrenal (HPA) axis. What about nonverbal evaluative feedback? Recent discoveries about the social functions of facial expression have documented three morphologically distinct smiles, which serve the functions of reinforcement, social smoothing, and social challenge. In the present study, participants saw instances of one of three smile types from an evaluator during a modified social stress test. We find evidence in support of the claim that functionally different smiles are sufficient to augment or dampen HPA axis activity. We also find that responses to the meanings of smiles as evaluative feedback are more differentiated in individuals with higher baseline high-frequency heart rate variability (HF-HRV), which is associated with facial expression recognition accuracy. The differentiation is especially evident in response to smiles that are more ambiguous in context. Findings suggest that facial expressions have deep physiological implications and that smiles regulate the social world in a highly nuanced fashion.

## Introduction

Sweaty palms, a racing heart, a faltering voice. Most people find the evaluative context of public speaking unpleasant. Indeed, the mere anticipation of social evaluation increases activity across almost all body systems related to stress, with particularly robust activation in the hypothalamic-pituitary-adrenal (HPA) axis^1–4^. However, scientific inquiry has largely been limited to investigations of the manner in which the body responds to verbal evaluative feedback, of the type “that was/wasn’t good.”^5,6^ Does the HPA axis respond to purely nonverbal feedback, such as facial expression? We investigate this question and demonstrate that evaluators’ smiles are sufficient to augment or dampen HPA axis activity – depending upon the distinct meaning of the smile in the social-evaluative context. Furthermore, we find that physiological responses to smile meaning are most differentiated in individuals with higher baseline high-frequency heart rate variability (HF-HRV), which is associated with facial expression recognition accuracy^7–9^.

Nonverbal feedback in social-evaluative contexts should be at least as impactful as verbal feedback, since nonverbal signals are experienced by perceivers to be spontaneous reactions^10^ and thus, honest reflections of internal evaluations. In the few studies that have investigated the nonverbal communication of evaluative feedback, participants whose audience displayed smiles and other nonverbal expressions of positive evaluation showed lower physiological activity than participants met with frowns and similar cues to negative evaluation^11–13^. Recent theory and empirical evidence suggests, however, that smiles do not all communicate identical, uniformly positive messages^14–16^. Instead, evidence supports the existence of at least three morphologically distinct types of smiles, each of which serves a different social function necessary for successful group living: “reward” smiles reinforce behavior, “affiliation” smiles signal lack of threat and facilitate or maintain social bonds, and “dominance” smiles assert claims to higher status in social hierarchies^15,16^. In light of their social functions, each of these three smiles should carry distinct meanings when displayed by evaluators. The first aim of the present research was to test the hypothesis that reward, affiliation, and dominance smiles, delivered as evaluative feedback, influence perceivers’ HPA axis activity in a manner congruent with their distinct social meaning. We expected dominance smiles, compared to reward smiles, to increase HPA axis activity. In theory, whereas dominance smiles show disdain for and reward smiles show approval of performance, affiliation smiles reassure without being indicative of a specific evaluation and so were expected to buffer HPA axis activity to a lesser degree than reward smiles.

The second aim of the present research was to account for variability in participants’ capacity to understand the evaluative meanings of each smile. Reward smiles are more unambiguous in meaning across contexts than either affiliation or dominance smiles^14^, which are relevant in more limited contexts. That is, reward smiles can reinforce widely varying behaviors in most any social situation, whereas affiliation smiles serve to smooth existing or potential social bonds and dominance smiles serve to challenge social standing. Individuals who are more accurate at recognizing facial expressions should exhibit more differentiated physiological activity in response to affiliation and dominance smiles, indicating greater sensitivity to their social-evaluative meanings. Recent empirical evidence suggests that baseline HF-HRV – an index of parasympathetic nervous system activity at the level of the heart – is positively associated with facial expression recognition accuracy^7–9^. We thus tested the hypothesis that individuals with higher baseline HF-HRV exhibit more differentiated physiological activity in response to smiles presented as evaluative feedback, particularly in response to smiles that are more ambiguous in context (i.e., affiliation and dominance smiles).

In an adaptation of the classic Trier Social Stress Test^17^, male participants (*N* = 90) extemporaneously addressed three topics about themselves in front of a male evaluator who watched them over a web camera. The evaluator was, in fact, one of two confederates working for the research team; approximately half of the participants (*N*s = 47 and 43) interacted with each confederate. To increase believability, the evaluator briefly appeared live on the computer screen, then turned off his web camera for the remainder of the session. After responding to each of the three topics, participants saw videos of their evaluator’s facial expressions, which they believed represented spontaneous reactions to their performance that had been extracted by facial recognition software. The videos were, in fact, pre-recorded. Participants were assigned to one of three smile conditions such that they saw either one reward, affiliation, or dominance smile after each of their responses to the three topics. Along with one smile video, participants also saw a control video that showed the evaluator making a neutral response such as face scratching or eye blinks. Thus, each participant was exposed to six videos of their evaluator in total (three different instances of one type of smile, three neutral videos) with one smile and one neutral video presented after each of the three responses. Smile videos were constructed to meet a priori specifications of morphological activity associated with each of the three smile types (for further details, see *Supplementary Materials*)^14^. Thus, smile type was the only feature of the evaluative feedback that varied between conditions. Physiological activity, in both the HPA axis and cardiovascular system, was assessed throughout the study; salivary cortisol was measured at seven time points, and a continuous electrocardiograph was collected before, during, and after the speech task.

## Results

Raw means for total salivary cortisol level by smile condition were consistent with the prediction that reward, affiliation, and dominance smiles influence perceivers’ physiological activity in distinctive ways, such that the receipt of dominance smiles is associated with higher HPA axis activity relative to the receipt of reward smiles. (AUC*i –* nmol/l: dominance: *M* = 19.4, *SD* = 24.74; affiliation: *M* = 2.43, *SD* = 22.3; reward: *M* = 1.21, *SD* = 21.54). We used dummy-coded condition contrasts to directly compare total salivary cortisol responses between smile types (“*AUCi*”, see Methods, below). Compared to reward smiles, dominance smiles induced a greater overall salivary cortisol response (*b* = 18.18, *t* (86) = 2.93, *p* = 0.004, *CI*(95%) = [5.83, 30.54], *Δr*^2^ = 0.087). Similarly, even though affiliation smiles do not signal a clear social evaluation in this context, compared to such smiles, dominance smiles induced a greater overall salivary cortisol response (*b* = 16.97, *t* (86) = 2.92, *p* = 0.004, *CI*(95%) = [5.41, 28.53], *Δr*^2^ = 0.086).

Further corroborating the finding that dominance smiles induce greater HPA axis activity, participants receiving reward or affiliation smiles returned to their individual cortisol baseline by 30-minutes post-speech, whereas those who received dominance smiles continued to have significantly higher cortisol levels than their individual baseline. In this ancillary analysis, cortisol values at 20- and 30-minutes post-speech were predicted from dummy-coded condition contrasts and average baseline cortisol level. Intercepts for each smile condition at both 20-minutes post-speech (reward, *b* = 0.23, *t* (86) = 1.49, *p* = 0.14, *CI*(95%) = [−0.08, 0.53]; affiliation, *b* = 0.25, *t* (86) = 1.79, *p* = 0.08, *CI*(95%) = [−0.03, 0.53]; dominance, *b* = 0.57, *t* (86) = 4.47, *p* < 0.0001, *CI*(95%) = [0.31, 0.82]) as well as 30-minutes post-speech (reward, *b* = −0.02, *t* (86) = −0.13, *p* = 0.9, *CI*(95%) = [−0.32, 0.28]; affiliation, *b* = 0.03, *t* (86) = 0.2, *p* = 0.84, *CI*(95%) = [−0.25, 0.31]; dominance, *b* = 0.36, *t* (86) = 2.82, *p* = 0.006, *CI*(95%) = [0.11, 0.61]) show that mean salivary cortisol values for the dominance group continued to be significantly greater than zero up to 30-minutes post-speech, which was not the case for the other groups. This shows that individuals receiving dominance smiles take significantly longer to return to their individual cortisol baseline, thus corroborating findings from the AUC*i* analysis.

**Figure 1:**
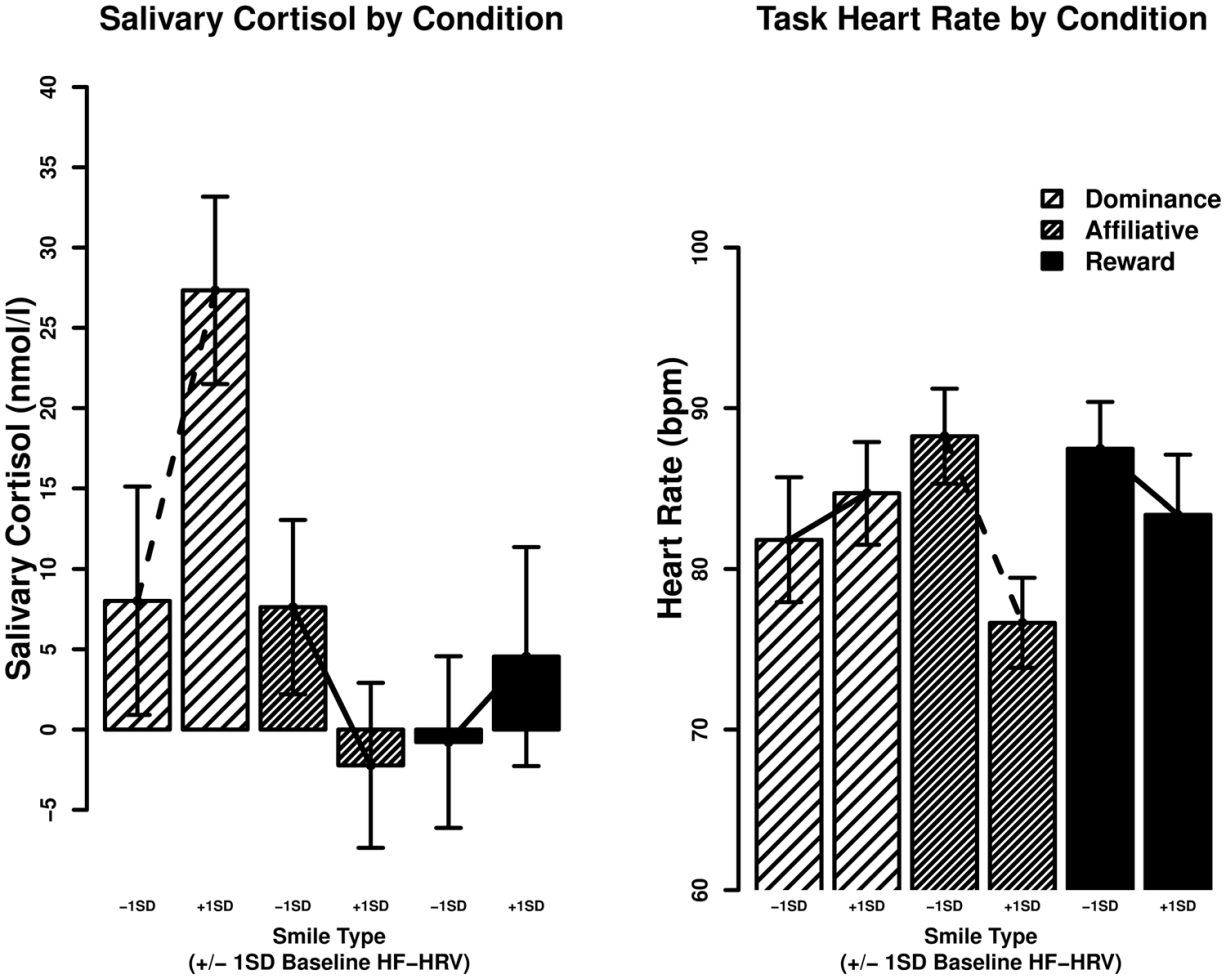
Physiological Response to Smiles as Evaluative Feedback Depends on Baseline High Frequency Heart Rate Variability. *Salivary Cortisol*: Total salivary cortisol (nmol/l: dominance: *M* = 19.4, *SD* = 24.74, *N* = 27; affiliation: *M* = 2.43, *SD* = 22.3, *N* = 36; reward: *M* = 1.21, *SD* = 21.54, *N* = 27) in response to social evaluation was greater for those receiving dominance smiles as evaluative feedback relative to the two other types of smiles. The difference in total salivary cortisol response between the affiliation and dominance groups increased as HF-HRV increased. *Heart Rate*: Heart rate assessed during the speech task (bpm: dominance: *M* = 83.51, *SD* = 9.84, *N* = 27; affiliation: *M* = 82.13, *SD* = 14.43, *N* = 36; reward: *M* = 85.93, *SD* = 12.65, *N* = 27) was not significantly different between conditions. The difference in heart rate between the dominance and affiliation groups increased as HF-HRV increased. Dotted lines between the +/− 1 SD bars indicate a statistically significant simple slope for baseline HF-HRV in that feedback condition; solid lines are not significant below the 0.05 level.

We also tested the prediction that baseline HF-HRV is positively associated with differentiation in HPA axis activity in response to affiliation and dominance smiles. We again created dummy-codes for smile feedback condition in order to compare the effect of smile feedback between conditions. We entered these dummy-codes along with a continuous measure of baseline HF-HRV and the dummy-code by HF-HRV interaction terms into a linear regression model. Results from this analysis confirmed our expectations. With those who received dominance smiles as the dummy-coded reference group, baseline HF-HRV was positively correlated with salivary cortisol responses (*b* = 9.72, *t* (84) = 2.02, *p* = 0.046, *CI*(95%) = [0.14, 19.29], *Δr*^2^ = 0.04). Comparing the linear association between baseline HF-HRV and salivary cortisol responses between individuals who received dominance versus affiliation smiles, the comparison of simple slopes was significant (*b* = 14.63, *t* (84) = 2.40, *p* = 0.018, *CI*(95%) = [2.54, 26.83], *Δr*^2^ = 0.057), indicating that the relationship between baseline HF-HRV and salivary cortisol is more positive for those receiving dominance compared to affiliation smiles as evaluative feedback (Fig. 1).

Convergent evidence that baseline HF-HRV is positively associated with greater differentiation of physiological responses to affiliation and dominance smiles comes from cardiovascular data. Again, we created dummy-coded condition contrasts and entered them into a linear regression model along with baseline HF-HRV and the HF-HRV by smile feedback dummy-code interaction terms. With the dummy-coded condition contrasts referenced on participants who received affiliation smiles, baseline HF-HRV was negatively associated with heart rate during the speech task (*b* = −5.84, *t* (84) = −2.85, *p* = 0.006, *CI*(95%) = [−9.91, −1.76], *Δr*^2^ = 0.086). A comparison of the linear association between baseline HF-HRV and heart rate between individuals who received affiliation versus dominance smiles reveals that the relationship between baseline HF-HRV and heart rate was more negative for those exposed to affiliation compared to dominance smiles as evaluative feedback (*b* = −7.28, *t* (84) = 2.19, *p* = 0.032, *CI*(95%) = [−13.92, −0.66], *Δr*^2^ = 0.051).

Since indicators of HF-HRV reactivity (i.e., baseline – task) have also been associated with facial expression recognition accuracy^18^, we conducted ancillary analyses of the association between reactivity measures of HF-HRV and perceivers’ physiological activity in response to smile feedback. Specifically, we calculated a difference score between task level HF-HRV and baseline by subtracting task HF-HRV, assessed during the first 2.5 minutes of the speech, from baseline HF-HRV. Higher values on this difference score reflect greater withdrawal of vagal influence over the heart during task, compared to baseline. We entered the difference score into a model similar to the one employed in the cortisol and heart rate analyses, entering dummy-coded condition contrasts along with the continuous HF-HRV difference score and the condition by difference score interactions. Table 1 reports these findings. HF-HRV reactivity was positively associated with physiological responses to social evaluation, regardless of the type of smile feedback a participant received (i.e., HF-HRV reactivity did not interact with experimental condition: see Table 1). These findings strengthen the conclusion that higher baseline HF-HRV is associated with more differentiated physiological responses to smiles as evaluative feedback.

**Table 1:** Associations Between HF-HRV Reactivity and Physiological Responses to Social Evaluation.

(N = 90)	*b* (s.e.)	vs. Affiliation	vs. Dominance
Reward			
Cortisol (AUC*i*)	7.04 (4.39)	0.95 (5.60)	1.30 (5.83)
Heart Rate	6.44** (2.31)	−0.32 (2.94)	−1.97 (3.06)
Affiliation			
Cortisol (AUC*i*)	7.99* (3.46)	—	0.36 (5.16)
Heart Rate	6.12** (1.82)	—	−1.65 (2.71)
Dominance			
Cortisol (AUC*i*)	8.35* (00.0)		—
Heart Rate	4.47* (2.01)		—

Unstandardized regression coefficients quantifying the association between HF-HRV reactivity (baseline – task) and physiological responses are reported in the left-hand column, for each group. Differences between groups are reported in the middle and right-hand columns. *p < 0.05; **p < 0.01; ***p < 0.001.

Analyses were conducted in the “R” statistical environment^19^. All results reported in the present work remain significant when adding a dichotomous predictor to account for which of the two confederates a participant interacted with, as well as when statistically accounting for factors known to influence levels of physiological activity (caffeine use, depression severity).

## Discussion

In the present study, we observed that smiles with different social functions^16^, when delivered as evaluative feedback in a stressful social context, exert distinct influences on perceivers’ physiological activity. Dominance smiles (compared to reward or affiliation smiles) were associated with increases in heart rate and salivary cortisol that mirror the influences of negative verbal feedback^20^. In contrast, reward and affiliation smiles exerted influences similar to the effects of displays of friendliness^21^ and positive social evaluation^12^, such that, compared to dominance smiles, they buffered physiological activity. The findings thus provide further evidence for the view that smiles do not constitute a homogenous category of “positive” nonverbal feedback. The findings also contribute to a body of literature that proposes a role for cortisol in adaptive responding to the social environment^22^. In particular, cortisol appears to support the detection of social threat and coordinate biological activity needed to adequately respond to the threat.

The present research also contributes to a growing literature on individual differences in sensitivity to the meaning of facial expression. Specifically, baseline HF-HRV was negatively related to participants’ physiological activity when they received affiliation smiles, and was positively related to their physiological activity when they received dominance smiles as evaluative feedback. The observed relationship between HF-HRV and physiological responsiveness to specific smile meaning is consistent with a claim of the Neurovisceral Integration Model according to which HF-HRV indexes an individual’s capacity to respond appropriately to social stimuli^23^. Conversely, individuals with particularly low baseline HF-HRV may be at risk of poor socio-emotional outcomes due to deficits in both understanding as well as responding to the social signals of others. A number of pathological and pre-disease body states are related to lower HF-HRV, including heightened inflammation and obesity^24,25^. As such, these body states may fundamentally change an individual’s ability to respond physiologically to the social world, and in light of the present findings, may be associated with deficits in understanding the expressions of others. A compelling avenue for future research therefore is to investigate how individuals with relatively low HF-HRV recognize and respond to the expressions of others.

Future research should also consider how affective disorders associated with lower HF-HRV, such as anxiety^26^ and depression^27^, relate to cognitive and physiological responses to social stimuli. Given the social stress procedure implemented in the present work, an especially fruitful avenue for future research on the social functions of smiles lies in the relationship between smile processing and social anxiety. Social anxiety disorder involves the fear of embarrassing oneself in front of others in performance or evaluative situations^28^. Although research consistently documents that individuals with social anxiety disorder exhibit heightened negative affect both in anticipation of and in response to social evaluation^29,30^, findings are mixed^31,32^ regarding how these individuals respond to positive and supportive social signals. Social anxiety was not accounted for in the present study. Although randomization into condition likely mitigated any effect of social anxiety on the present findings, future work is needed to investigate how individuals with social anxiety respond to facial expressions as evaluative feedback. A crucial comparison in this work will be to assess differences in affective and physiological responses between the relatively unambiguous reward smiles and the less clear affiliation smiles.

One limitation of the present research is that the experimental design contained no “neutral” feedback condition. It would be hard to create such an experience, since neutral feedback is interpreted in highly varied ways across individuals. However, methods to create such a control condition could be imagined for future research. Along similar lines, future research could also directly compare the effects of receiving dominance smiles with the effects of receiving more overt signals of negative evaluation such as disgust, contempt, and anger. By employing the present paradigm to test the physiological effects of facial expressions beyond the smiles tested here, researchers could not only more clearly describe the unique effects of receiving social functional smiles but also understand the effects of facial expressions on perceivers’ physiological activity more generally.

The present work includes other limitations that warrant comment, two of which concern the participant sample. First, the sample size in this study may have been relatively small given the size of the effects detected. Since, to our knowledge, no work has explored the effect of receiving social functional smiles as social evaluation, a priori power analyses were difficult to conduct. We estimated required sample size from extant studies with methods and aims as similar to the present research as possible^12^. Following these guidelines, 90–100 participants is typical for a study of this nature involving three between-subjects conditions. Post-hoc power analyses using G*Power^33^ indicate that achieved power was modest for the critical comparisons within the HPA axis (dominance vs. affiliation: β = 0.72; dominance vs. reward: β = 0.81). In light of the achieved power, we recommend that future studies of this sort rely on no fewer than 35–40 participants per between-subjects condition to ensure adequate power.

Second, the present sample was restricted to men. Although we limited the sample to males for reasons that were established and justified before the research was conducted (see Methods, below), generalizations from our findings are necessarily limited. Since sex effects are observed in research on the perception of facial expression of emotion^34,35^, future work should determine what portion (if any) of the currently documented effects are contingent upon the sex of the smiler or perceiver. Some work suggests that men respond more to threats of physical aggression whereas women respond more to condescending behaviors and social aggression^36^. Thus, men and women may respond to the same type of smile in different ways and may also use each of the smile types with varying degrees of frequency^37^.

The present research demonstrates that functionally different smiles are sufficient to augment or dampen HPA axis activity in accordance with the social functional meaning associated with each smile. Furthermore, physiological responses to each functionally distinct smile are most differentiated in individuals with higher baseline HF-HRV, suggesting that HF-HRV is a useful individual difference moderator of the ability to respond to the social environment in a flexible and nuanced fashion. We may therefore conclude that smiles coordinate the physiological activity that supports interpersonal encounters to a previously undocumented degree, and that facial expressions help regulate the social world, in part, through their impact on the physiological activity of perceivers.

## Methods

### Participants

Ninety-two male undergraduates at a large university in the Midwest participated in exchange for credit in an introductory Psychology course. Participants provided written consent, indicating full understanding of the requirements for participation. The research protocol was reviewed and approved under the University of Wisconsin – Madison Institutional Review Board. All research was conducted in accordance with institutional guidelines and regulations.

Due to the robust sex differences in cortisol responses to laboratory stressors^38^ and the variability in cortisol responses introduced by oral contraceptive use^39^, only males were invited to participate in this study. Since females generally outperform males in the accurate recognition of positive facial expressions^40^, the expectation was that the present study would underestimate the effects of smiles on physiological responses. Pre-inclusion criteria limited participation to U.S.-born, English-speaking males without a diagnosed heart condition and not currently taking medications that alter hormone levels. Participants were instructed to refrain from exercise on the day of the study and to avoid alcohol and caffeine consumption within twenty-four hours of their participation. Due to a network failure, data collection from one participant was terminated before experimental manipulation. Furthermore, data from a second participant were excluded from analysis due to the presence of an abnormal heart rhythm resembling premature beats^41^ which made it difficult to score the data and conflicted with the pre-inclusion criterion of cardiac health. The exclusion of all data from these two participants left a final sample of ninety participants (dominance: *N* = 27; affiliation: *N* = 36; reward *N* = 27).

Data collection was limited to the number of participants that could be involved during one academic semester, not to exceed 120 participants (40 per condition). Participants were randomly assigned to one of three smile experimental conditions as well as one of two confederates. Given that some participants did not show up for their assigned experimental sessions, a certain amount of imbalance between the number of participants in each condition and assigned to each confederate is to be expected.

At the conclusion of the study, participants underwent a funneled debriefing. First, they were asked if they thought they knew what the study was about. In this general interview, no participants brought up suspicions about deception. We then asked participants if they found anything strange about the study or were suspicious of anything. In a logistic regression model with experimental condition and confederate as predictors of a dichotomous (“yes”/“no”) suspicion outcome, no significant differences were detected by experimental condition (all *p*s > 0.2) or by confederate (*p* = 0.93). Participants who thought the study was slightly strange indicated that they were unsure how the filler video was related to the study, were unaccustomed to providing saliva samples, or were uncomfortable giving speeches.

### Procedure

In order to reduce variation due to diurnal changes in cortisol levels, experimental sessions took place in the afternoon. Upon arriving at the experimental laboratory, the participant encountered another male “participant” who was actually a confederate—one of the two whose stimuli were validated in a separate study (see *Supplemental Material* for further information). The experimenter then entered from a nearby room and told the two men that he had randomly assigned them to different tasks in the study: the participant was always assigned to “give the speech” and the confederate was always assigned to “judge the speech.” After the participant and confederate had provided informed consent, the participant supplied the first of seven saliva samples (collection method described below) and completed an online questionnaire assessing his compliance with pre-restriction criteria (alcohol and caffeine use) as well as other medical information (prescription and recreational drug use). The men were told that only the person giving the speech (always the participant and never the confederate) had to answer the online questionnaires and provide the saliva samples because it directly pertained to giving the speech; the confederate waited with the participant and the experimenter while the participant responded to the surveys and provided the saliva sample.

The experimenter then demonstrated a facial expression recognition software, the Computer Emotion Recognition Toolbox^42^. The participant and confederate were informed that the program could extract meaningful facial expressions from live video feed. The experimenter used the computer’s built-in web camera and the participant’s face as the live feed in a demonstration in order to increase believability in the software’s (actual) capabilities.

Next, the participant and confederate were separated. Leaving the confederate behind in the initial room, the experimenter mentioned that a second experimenter would arrive shortly to provide the confederate with further instructions and tasks. The experimenter escorted the participant to a psychophysiology lab in the same building. There, the experimenter attached sensors to the participant’s chest and explained that they would be used to measure aspects of his cardiovascular reactivity. The experimenter sat with the participant in the experimental room while a research assistant in an adjacent control room recorded a 3-minute baseline measure of the participant’s heart activity.

After completion of this baseline recording, the experimenter informed the participant that he would deliver his speech to the “other participant” via Skype. He would not see the evaluator as his web camera would be turned off in order to avoid distraction. The format of the speech involved answering three questions in sequence, with two minutes to respond to each question. The participant was also told that at the conclusion of every two-minute speech period, he would see videos of several of the evaluator’s facial expressions. The participant was told that the videos had been “randomly extracted by the facial expression software” while the evaluator watched the participant’s speech. This led the participant to believe that the videos conveyed authentic evaluative responses by the confederate. The facial expressions displayed in these videos constituted the experimental manipulation.

When the participant indicated that he had understood the speech task, the experimenter gave him a sheet of paper with the three questions he was required to answer, and then sat with the participant for three minutes while the participant prepared his speech. A continuous cardiovascular recording was taken during this three-minute “anticipation” period. After the time was over, the second saliva sample was taken.

Next, the experimenter launched Skype. In order to enhance believability, the confederate appeared live on the participant’s screen and waved “hello.” The experimenter asked the evaluator to turn off his camera “so as not to distract” the participant during the speech. With the participant no longer able to see the evaluator, the experimenter asked the participant to begin his speech. Participants responded to each question in order, with two minutes for each question: (1) What makes you happy? (2) What do you like most and least to eat?, and (3) What is your favorite part of living in Madison, Wisconsin? These questions were designed to be personal and to contain enough positive material to make the smiles sent by the evaluator appear plausible.

At the conclusion of each two-minute speech period, the experimenter stopped the participant and showed him two videos of the evaluator that were “randomly extracted” when he was listening to the participant’s speech. From the other room, the confederate dropped each video into a network-based folder in order to simulate the facial expression recognition program extracting and sending the videos in real time. Each participant saw 6 videos in total, 2 after each question. 3 of the 6 total videos were smiles and the other 3 were a set of different neutral videos—evaluators faced the camera with a neutral expression and made occasional small, non-evaluative movements such as face scratching. The smile videos were three different short video clips of the same confederate making only one smile type (dominance, reward, or affiliation). Participants were thus exposed to three different examples of the same type of smile. In sum, smile type was manipulated between-subjects with the 3 neutral videos retained across participants. At the conclusion of the speech task, the third saliva sample was taken. The speech task lasted between 7–8 minutes, during which a continuous cardiovascular recording was taken.

Immediately upon concluding his final speech, the participant was directed to reflect on his performance, focusing on how he felt and what his evaluator thought. A continuous cardiovascular recording was taken during this five-minute reflection period. After the reflection/recovery period, the participant was detached from the sensors and led to the final room by a second experimenter who was blind to the participant’s video feedback condition.

In the last room, the participant watched a filler video available on YouTube from the series “The Life of Birds” (available at https://www.youtube.com/playlist?list=PLB1F251E81DE15E9B) which provided a neutral experience during which cortisol recovery was assessed. The video was the same for all participants. The remaining four saliva samples were taken at 10-minute intervals from the cessation of the speech task. At the conclusion of the filler video, and after completing verbal questions assessing deception suspicion, the participant was debriefed and dismissed.

### Physiological Measures: Collection and Analysis

#### Cortisol

Saliva samples were obtained with cotton salivettes (Fisher Scientific Company, LLC). Participants were instructed to let the cotton salivette touch all parts of their mouth (under their tongue, between their teeth and their cheeks) without chewing on it. Saliva collection was strictly timed for two minutes, after which the sample was returned to its plastic casing. Samples were frozen after collection and stored at −20 C. At the conclusion of the study, samples were express shipped to Dresden, Germany where they were single-assayed at the lab of Dr. Clemens Kirschbaum (T.U. Dresden). Samples were assayed using the chemi-luminescence assay, which has a high sensitivity of 0.16 ng/mL (IBL-International, Hamburg, Germany) and intra and interassay CVs of <10%. In total, saliva samples were collected at seven time points during the study and assayed for unbound cortisol. Due to skewness, all cortisol values were first log-transformed. Salivary alpha amylase was assayed but is not reported in these analyses.

In order to test our hypotheses with regard to the HPA axis, Area Under the Curve with respect to increase (AUC*i*)^43^ values were calculated for the cortisol response of each participant. AUC*i* scores index total cortisol response over a given period of time, referenced to each individual’s baseline cortisol level. We averaged the two cortisol values collected before experimental manipulation (receipt of smile feedback) as a pre-speech baseline.

#### Heart Rate

Continuous EKG recordings were sampled at 1000 Hz via one of the bipolar inputs available on the SynAmps2 Headbox (Compumedics Neuroscan Ltd., U.S.A). Ag/AgCl spot electrodes were placed in a thoracic-modified lead-II configuration to maximize detection of R-spikes while minimizing movement artifacts. We calculated mean heart rate values separately for the baseline period, the “anticipation” period, the speech period, and the post-speech period.

#### Data Processing

EKG data were first scored offline using OpenANSLAB^44^, manually inspected for artifacts, and the resultant inter-beat-interval series were extracted and saved. CMETx software (available at http://apsychoserver.psych.arizona.edu) was then used on the extracted inter-beat-interval to quantify HF-HRV.

## Electronic supplementary material

Supplementary Materials
