# Supplementary material for: Functionally distinct smiles elicit different physiological responses in an evaluative context
Source: Sci Rep. 2018 Mar 1;8:3558. doi: 10.1038/s41598-018-21536-1 (PMC5832797; doi:10.1038/s41598-018-21536-1)
Supplement: Supplementary file 1 — Supplementary Materials [file 41598_2018_21536_MOESM1_ESM.docx]

Functionally distinct smiles elicit different physiological responses in an evaluative context

Jared D. Martin^a*^, Heather C. Abercrombie^b^, Eva Gilboa-Schechtman^c^, & Paula M. Niedenthal^a^

aUniversity of Wisconsin-Madison, Department of Psychology, 1202 W. Johnson St., Madison, WI, 53706, United States. bUniversity of Wisconsin-Madison, Department of Psychiatry, 6001 Research Park Blvd, Madison, WI, 53719, United States. cBar-Ilan University, Department of Psychology, Ramat-Gan, 52900, Israel. Correspondence and requests for materials should be addressed to J.D.M. (email: jdmartin7@wisc.edu)

**Supplemental Materials: Smile Stimuli Creation and Validation**

The smile stimuli from our two confederates were created with the social evaluation task in mind, and we therefore ensured that the stimuli reflected the social situation (i.e., listening to a speech over Skype) to the extent possible. To enhance believability, the confederates were filmed in the same room in which they would eventually meet the participants during the experiment, and from the same angle and distance as the web camera through which they would briefly appear on Skype. Confederates wore the same clothing during every session. Results from a separate study validating that each of the three smile types communicates the desired social-functional meaning are reported below.

*Participants.* 166 participants (*M* (age) = 36.5, 60% male) were recruited from Amazon’s Mechanical Turk web service. As a requirement for eligibility, all participants resided in the United States at the time of data collection.

*Facial Expression Videos.* Informed by models of the three functional smiles derived from a data-driven approach^14^ we created a set of facial expression videos. Two individuals, who were to serve as confederates in the social evaluation task, were coached to imagine themselves in contexts in which the three smiles (reward, dominance, affiliation) might be encountered and to make a smile that would be displayed in that context. Only in the event that the confederates did not achieve a facial expression resembling the models of Rychlowska and colleagues^14^ were they given instructions to involve certain facial muscles in the expressions. In addition, the facial expressions of sadness, disgust, anger, positive surprise, and neutrality were also recorded.

We selected three videos of each smile type (nine total), three neutral expressions, and one each of expressions of sadness, positive surprise, anger, and disgust from the two confederates. This resulted in a total of 32 videos for use in the present study. In order to ensure that the smiles were morphologically adequate representations of the smile animations used in previous studies^14^, we visually examined the stimuli with a computer-assisted facial expression coding platform^42^. Rychlowska and colleagues^14^ document that Action Units 1-2 are related to reward smiles, AU 14 to affiliation smiles, and asymmetrical activation of AU12 to dominance smiles. Visual inspection shows that each smile type is morphologically distinct in physical features outlined by previous research^14^.

*Procedure*. The online survey involved three tasks, which were always presented in a single order: Smile Categorization, Signal Rating, and Message Choice.

*Smile Categorization Task.* Facial expression videos were each presented once, for a total of 32 trials. On every trial, participants indicated whether the facial expression was a smile or not, by selecting the label "yes" or "no" with the mouse. The order of video presentation was randomly determined for each participant.

*Signal Rating Task.* Upon completion of the smile categorization task, participants were randomly assigned to one of 3 between-subjects rating conditions (happy/good: *N* = 54; approachable/cooperative: *N =* 56; superior/dominant: *N* = 56). According to condition assignment, participants rated all 18 smile stimuli on a single meaning dimension from 1 “not at all” to 7 “very much”: "The expression means the person is feeling good/happy" (reward); "The expression means the person is feeling approachable/cooperative " (affiliation); “The expression means the person is feeling dominant/superior" (dominance). The 18 smile videos were presented in random order.

*Message Choice Task.* In the final task, participants again saw all 18 smile videos. They were asked to imagine that they had just given a speech in front of the person in the video. Their task was to select the message that best matched the meaning of the facial expression in the video. Participants chose from 7 response options. Three options were evaluative, and related to the functional smiles as follows: “Your speech was good,” (reward); “Your speech was okay,” (affiliation); “Your speech was bad” (dominance). Three options were written to approximate the functional message communicated by the smile types in a speech context: “Hey, I liked what you said,” (reward); “Good try, I know it's hard,” (affiliation); “I could have done better” (dominance). Participants were also given the option to choose “None of the above.” The 18 smile videos were presented in random order.

*Statistical Analysis and Results*: *Smile Categorization Task.* All analyses were conducted in the “R” statistical environment^19^. First, we collapsed smile/not smile categorizations across both confederates and all instances of each stimulus type. The left-hand portion of **Figure S1** depicts percentage categorization for each of the eight stimulus categories. In order to analyze whether participants were more likely to categorize instances of smile stimuli as smiles versus not smiles, we fit a logistic regression model with a dummy-coded stimulus factor representing the eight stimulus categories. In this model, a significant intercept value indicates that mean categorization rates were significantly different from chance (50%) for the “reference group” dummy-coded stimulus category. Re-referencing the stimulus factor on each of the eight expressions reveals that all three smile expressions were more likely to be seen as smiles than not (reward: *b =* 4.59, *CI*95% = [4.02, 5.28], *z* = 14.44, *p* < .0001; affiliation: *b =* 3.37, *CI*95% = [3.04, 3.74], *z* = 19.05, *p* < .0001; dominance: *b =* 1.31, *CI*95% = [1.06, 1.46], *z* = 16.89, *p* < .0001). Conversely, all five other expressions were more likely to be categorized as not smiles than as smiles (Neutral: *b =* -3.83, *CI*95% = [-4.3, -3.43], *z* = -17.4, *p* < .0001; surprise: *b =* -1.83, *CI*95% = [-2.01, -1.65], *z* = -19.93, *p* < .0001; anger: *b =* -1.84, *CI*95% = [-2.02, -1.66], *z* = -19.96, *p* < .0001; sadness: *b =* -1.81, *CI*95% = [-2.0, -1.64], *z* = -19.86, *p* < .0001; disgust: *b =* -4.05, *CI*95% = [-4.57, -3.61], *z* = -16.57, *p* < .0001). Results remain significant when accounting for multiple ratings from participants via multi-level logistic regression analyses^45^. Furthermore, analyses including participant sex as a factor in both the original analysis as well as in a multi-level logistic regression model did not reveal sex as a significant predictor in any categorization (all *p*s > .9).

*Statistical Analysis and Results*: *Signal Rating Task.* 3 between-subjects groups rated all smile stimuli on one of three dimensions (happy/good: *N* = 54; approachable/cooperative: *N =* 56; superior/dominant: *N* = 56). We analyzed ratings separately by stimulus type. As expected, reward smiles received higher “happy/good” ratings (*M=*6.39, *SD*=0.62) than they did “approachable/cooperative” ratings (*M=*6.29, *SD*=0.76) or “dominant/superior” ratings (*M=*3.1, *SD*=1.54). Using orthogonal contrasts, we compared “happy/good” ratings to “approachable/cooperative” and “dominant/superior” (contrast: 1, -.5, -.5), also including the further comparison “approachable/cooperative” to “dominant/superior” (contrast: 0, .5, -.5) in order to account for residual variance. Results indicated that reward smiles received significantly higher “happy/good” ratings compared to other ratings (*b* = 2.26, *t* (163) = 9.68, *p* < .0001, *CI*(95%) =[1.8, 2.72], *Δr^2^* = .18) and were viewed as more “approachable/cooperative” than “dominant/superior” (*b* = 3.19, *t* (163) = 15.0, *p* < .0001, *CI*(95%) =[2.8, 3.58], *Δr^2^* = .50).

As expected, affiliation smiles received higher “approachable/cooperative” ratings (*M=*5.13, *SD*=0.93) than “happy/good” (*M=*5.0, *SD*=0.97) or “dominant/superior” ratings (*M=*3.95, *SD*=1.15). Using orthogonal contrasts, we compared “approachable/cooperative” ratings to “happy/good” and “dominant/superior” (contrast: 1, -.5, -.5) also including the further comparison “happy/good” to “dominant/superior” (contrast: 0, .5, -.5). Results indicated that affiliation smiles received significantly higher “approachable/cooperative” ratings compared to the other ratings (*b* = 0.88, *t* (163) = 4.24, *p* < .0001, *CI*(95%) =[0.47, 1.29], *Δr^2^* = .08) and were viewed as more “happy/good” than “dominant/superior” (*b* = 1.05, *t* (163) = 5.82, *p* < .0001, *CI*(95%) =[0.69, 1.41], *Δr^2^* = .16). Dominance smiles received higher “superior/dominant” ratings (*M=*5.44, *SD*=0.97) than “happy/good” (*M=*4.50, *SD*=0.89) or “approachable/cooperative” ratings (*M=*4.11, *SD*=1.15). Using orthogonal contrasts, we compared “superior/dominant” ratings to “happy/good” and “approachable/cooperative” ratings (contrast: 1, -.5, -.5), again including the further comparison “happy/good” to “approachable/cooperative” (contrast: 0, .5, -.5) in order to account for residual variance. Results indicated that dominance smiles received significantly higher “superior/dominant” ratings compared to the other ratings (*b* = 1.51, *t* (163) = 6.82, *p* < .0001, *CI*(95%) =[1.07, 1.95], *Δr^2^* = .22) and were viewed as more “happy/good” than “approachable/cooperative” (*b* = 0.34, *t* (163) = 2.05, *p* = 0.42, *CI*(95%) =[0.02, 0.78], *Δr^2^* = .02).

*Statistical Analysis and Results*: *Message Choice Task.* We first collapsed categorizations across both the evaluative and functional messages (i.e., dominance: “I could have done better” or “Your speech was bad”) as well as all instances of each stimulus type for both confederates. The right-hand confusion matrix in **Fig. S1** depicts percentage of response choice for the three stimulus types. Comparison of stimulus category to perceivers’ message choice using Cohen’s κ, a measure of inter-rater reliability, revealed κ = .45, *CI*95%[.42, .48], which is considered “moderate” by traditional cut-off values^46^.

In order to analyze whether participants were more likely to choose a message label corresponding to one of the two matching messages versus a non-matching message, we employed a similar analysis strategy as in the smile categorization task. We used the “VGAM” package^47^ in R to fit a multinomial logistic regression model (outcomes: “None of the above,” “reward message,” “dominance message,” “affiliation message”) with a dummy-coded stimulus factor representing the 3 smile categories. Re-referencing the stimulus factor on each of the three expressions allows for a comparison of matching message choice to the three alternative choices. Results indicate that participants were more likely to choose the matching message than any other response, for all smile types: reward (reward message vs. affiliation message: *b =* -1.7, *CI*95% = [-1.87, -1.52], *z* = -19.04, *p* < .0001; reward vs. dominance: *b =* -3.44, *CI*95% = [-3.83, -3.05], *z* = -17.28, *p* < .0001; reward vs. “none of the above”: *b =* -6.01, *CI*95% = [-7.4, -4.62], *z* = -8.49, *p* < .0001), affiliation (affiliation message vs. reward message: *b =* -0.48, *CI*95% = [-0.63, -0.34], *z* = -6.56, *p* < .0001; affiliation vs. dominance: *b =* -0.91, *CI*95% = [-1.07, -0.74], *z* = -10.69, *p* < .0001; affiliation vs. “none of the above”: *b =* -3.54, *CI*95% = [-4.07, -3.01], *z* = -13.06, *p* < .0001), dominance (dominance message vs. reward message: *b =* -1.88, *CI*95% = [-2.11, -1.65], *z* = -16.25, *p* < .0001; dominance vs. affiliation: *b =* -0.56, *CI*95% = [-0.7, -0.42], *z* = -8.03, *p* < .0001; dominance vs. “none of the above”: *b =* -3.44, *CI*95% = [-3.91, -2.98], *z* = -14.4, *p* < .0001).


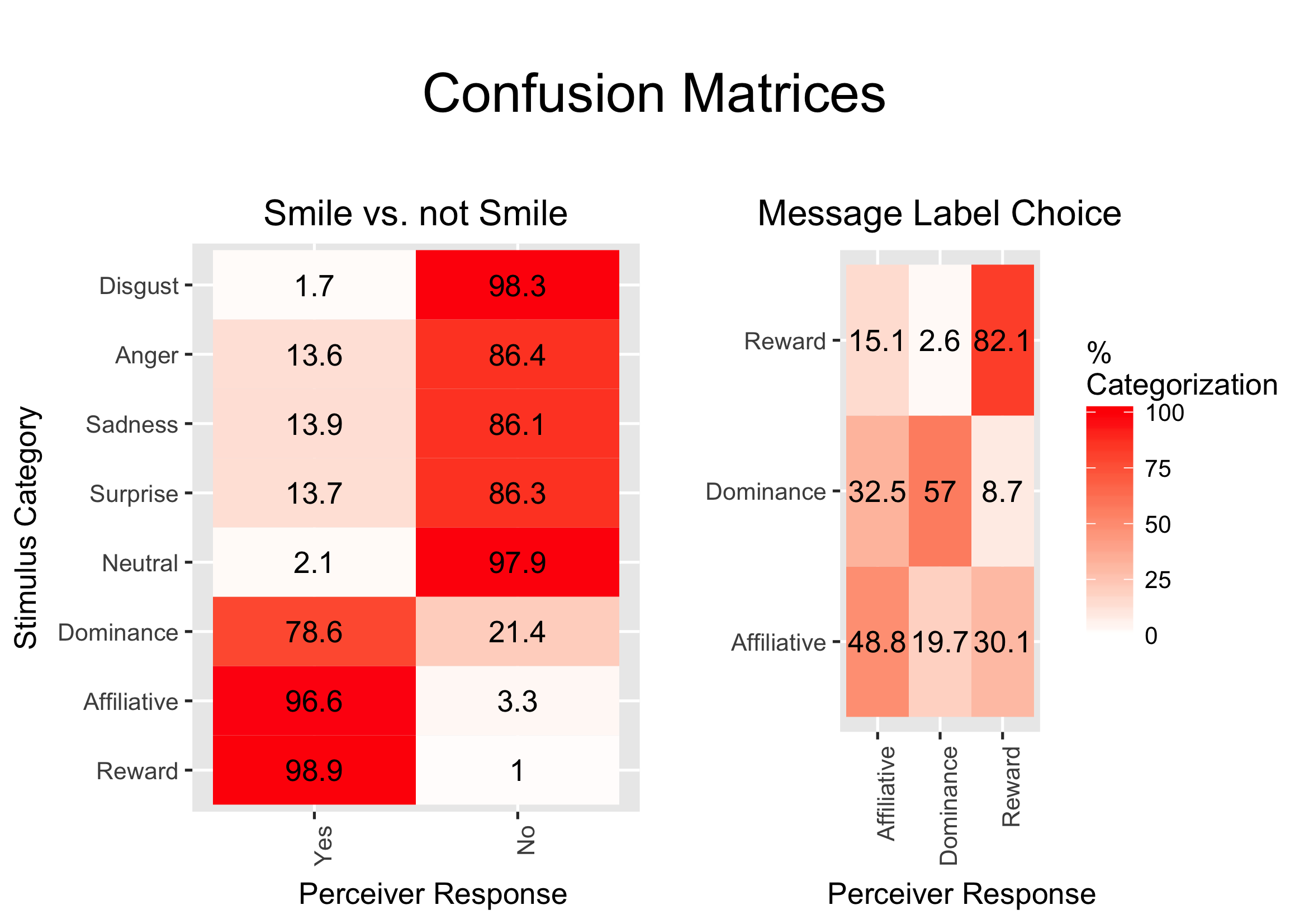


**Fig S1***.* **Confusion Matrices for Stimulus Construction.** Confusion matrices depict the outcomes of Smile Categorization Task (left) and Message Choice Task (right). For the right-hand confusion matrix, note that “None of the above” was factored into the percentages but omitted from the figure.

**References**

1. Bates, D., Maechler, M., Bolker, B. & Walker, S. Fitting linear mixed-e ects models using lme4. J. S*tat*. S*o* . **67** (2015).
2. Landis, J. R. & Koch, G. G. e measurement of observer agreement for categorical data. *Biometrics* **33**, 159–174 (1977).
3. Yee, T. W. & Wild, C. J. Vector generalized additive Models. *J. R. Stat. Soc. Ser. B* **58**, 481–493 (1996).
